# A 2x2 randomised factorial SWAT of the use of a pen and small, financial incentive to improve recruitment rates in a randomised controlled trial of yoga for older adults with multimorbidity

**DOI:** 10.12688/f1000research.52164.1

**Published:** 2021-04-27

**Authors:** Caroline Fairhurst, Jenny Roche, Laura Bissell, Catherine Hewitt, Jess Hugill-Jones, Jenny Howsam, Camila S Maturana, Belen Corbacho Martin, Shirley-Anne S Paul, Fi Rose, David J Torgerson, Lesley Ward, Laura Wiley, Garry A Tew

**Affiliations:** 1 York Trials Unit, ARRC Building, Department of Health Sciences, University of York, Heslington, York, YO10 5DD, UK; 2 British Wheel of Yoga Qualifications, 25 Jermyn Street, Sleaford, NG34 7RU, UK; 3 Department of Sport, Exercise and Rehabilitation, Northumbria University, Newcastle upon Tyne, NE1 8ST, UK

**Keywords:** study within a trial, pen, financial incentive, recruitment, factorial, randomised controlled trial, older people, multimorbidity

## Abstract

**Background:** Monetary and other incentives may increase recruitment to randomised controlled trials.

**Methods: **This was a 2x2 factorial ‘study within a trial’ of including a pen and/or £5 with a postal recruitment pack to improve randomisation rate (primary outcome) into the host Gentle Years Yoga trial in older adults with multimorbidity. Secondary outcomes: return, and time to return, of screening form, and the cost per additional participant recruited. Binary data were analysed using logistic regression and time to return data using Cox proportional hazards regression.

**Results: **818 potential host trial participants included. Between those sent a pen (n=409) and not sent a pen (n=409), there was no evidence of a difference in the likelihood of being randomised (15 (3.7%) *versus* 11 (2.7%); OR 1.38, 95% CI 0.63–3.04), in returning a screening form (66 (16.1%) *versus* 61 (14.9%); OR 1.10, 95% CI 0.75–1.61) nor in time to return the screening form (HR 1.09, 95% CI 0.77–1.55). There was evidence of improved screening return rates (77 (18.8%) *versus* 50 (12.2%); OR 1.67, 95% CI 1.13–2.45) and time to return screening form (HR 1.56, 95% CI 1.09–2.22) but not randomisation (14 (3.4%) *versus* 12 (2.9%); OR 1.18, 95% CI 0.54–2.57) in those sent £5 (n=409) compared with those not sent £5 (n=409). No significant interaction effects between the interventions were observed. The cost per additional participant recruited was £32 for the pen and £1000 for the £5 incentive.

**Conclusion: **Including a small, monetary incentive encouraged increased and faster response to the recruitment invitation but did not result in more participants being randomised into the host trial. Since it is relatively costly, we do not recommend this intervention for use to increase recruitment in this population. Pens are cheaper but did not provide evidence of benefit. Further studies may be required.

## Introduction

Efficient recruitment to randomised controlled trials (RCTs) is important to achieve the target sample size and statistical power within the planned budget and time frame. Incentives, monetary or otherwise, are sometimes used to increase trial recruitment. ^1^ We conducted a ‘study within a trial’ (SWAT) to evaluate the effects of including a small, unconditional financial incentive and/or a pen in the postal recruitment pack on the rate of randomisation into the host trial.

## Methods

### Design

This 2x2 randomised factorial SWAT was embedded in the Gentle Years Yoga (GYY) trial, which is a multi-centre RCT of the clinical and cost-effectiveness of a yoga programme for older adults with multimorbidity (currently recruiting; ISRCTN13567538, registered 18/03/2019 https://www.isrctn.com/ISRCTN13567538). The SWAT was registered with the Northern Ireland Network for Trials Methodology Research SWAT repository on 01/04/2018 ( SWAT94; https://www.qub.ac.uk/sites/TheNorthernIrelandNetworkforTrialsMethodologyResearch/SWATSWARInformation/Repositories/SWATStore/). The GYY trial, and its embedded sub-studies, has approval from the North East–York Research Ethics Committee, obtained on 24/04/2019 (19/NE/0072), and the Health Research Authority.

### Participants

Patients who appeared to meet the GYY trial eligibility criteria and were to be mailed a recruitment pack, as identified by four participating GP practices, were eligible for inclusion in this methodological sub-study. The recruitment pack included an invitation letter, participant information sheet, consent form, screening form, and prepaid envelopes to return documentation to the York Trials Unit, University of York. A random sample of packs also included a £5 note and/or a pen (branded with the trial logo) as part of this SWAT. The packs were sent out in August 2019.

### Interventions

Financial incentives have been found to increase recruitment by 4% (95% CI -1–8%) in a meta-analysis. ^1^ However, most of the included studies used an incentive of £100, which is larger than publicly funded trials can usually afford. There remains, therefore, uncertainty as to whether financial incentives should be used and, if so, what amount.

Offering a potential participant a gift such as a pen may make them more likely to take up the invitation to enrol in a trial. It is also possible that the convenience of having a pen to hand upon receipt of the invitation may help facilitate a swifter response. However, a previous SWAT conducted by the York Trials Unit evaluated the use of pens as an incentive for recruitment into the OTIS trial of older adults and showed no difference on randomisation rate (pen 4.5%; no pen 4.3%, odds ratio (OR) 1.04, 95% CI 0.65–1.67, p = 0.86), screening rate (pen 14.2%, no pen 11.7%, OR 1.25, 95% CI 0.94–1.67, p = 0.12), or time to return screening form (hazard ratio (HR) 1.23, 95% CI 0.94–1.60, p = 0.13). ^2^ To our knowledge, this is the only previous RCT to evaluate pens to increase trial recruitment, so more evidence is needed; hence, we conducted this SWAT.

### Outcome measures

The primary outcome was randomisation into the host GYY trial. Secondary outcomes were return, and time to return, of a screening form to the York Trials Unit. The cost per additional participant recruited was calculated for each intervention.

### Sample size and randomisation

Due to financial restrictions, we could afford to involve a sample of 850 recruitment packs in this SWAT. This would give 80% power (two-sided α=0.05) to detect a difference in recruitment rate of 4% (from 3% to 7%) for either of the interventions, relative to not receiving that intervention.

Block randomisation of size 4 was used to allocate recruitment packs 1:1:1:1 to: no pen or £5; £5 only; pen only; or pen and £5. A trial statistician, not involved in the production of recruitment packs or recruitment of participants, generated the sequence using Stata v15 (RRID: SCR_012763). Stata is a proprietary software but an open-access alternative in which the sequence could have been generated is Microsoft Excel (RRID: SCR_016137).

### Blinding and consent

The statisticians were not blinded to allocation. Similarly, due to the nature of the interventions, participants could not be blinded to their allocation; however, they were not specifically informed about the SWAT nor that the incentive they received had been chosen through a process of randomisation. Specific consent for the trial was not required by the Research Ethics Committee, as it was considered low risk. Written informed consent for the main trial was obtained from all participants who agreed to take part in GYY.

### Statistical analysis

The primary comparisons in this trial are the main effects of being sent a pen, and of being sent a £5 note. Returning a screening form and being randomised into the GYY trial were both analysed using multivariable logistic regression, including the two interventions (pen and £5). Time to return the screening form (in days from the date the recruitment pack was sent out to the date it was returned) was analysed using Cox proportional hazards regression. Screening forms that were not returned were censored at eight weeks after they were sent out. These analyses provide an estimate of the average effect of each intervention, assuming there is no interaction between them. In secondary analyses, the interaction between the two interventions was tested by extending the original models to include the interaction term. Analyses were conducted in Stata v16 (RRID: SCR_012763). An open-access alternative that can perform an equivalent function to Stata for analysis is R, a free software environment for statistical computing and graphics (RRID: SCR_001905).

## Results

**Figure 1.**
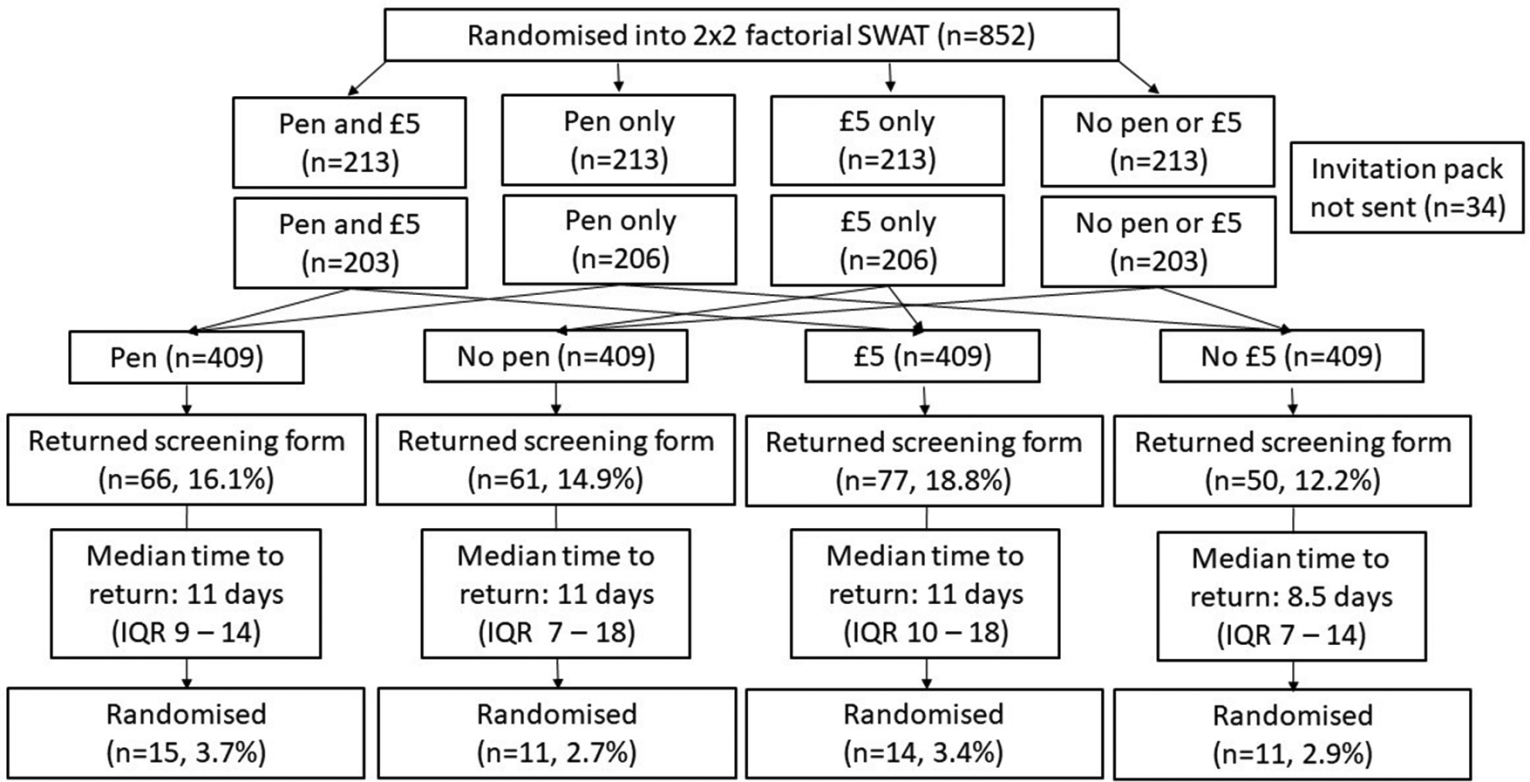
Participant flow diagram.

In total, 852 allocations were generated but, due to one of the participating GP practices having a shorter mailing list than anticipated, only 818 (96.0%) were used ( Table 1; Figure 1). In these analyses, the potential participants who were sent a pen (n = 409) consist of the group who received a pen and a £5 note (n = 203), and the group who received a pen only (n = 206). These are compared with those who were not sent a pen (n = 409), consisting of the group who were only sent a £5 note (n = 206), and the group who were sent neither a pen nor £5 (n = 203). Similarly, those who were sent £5 (n = 409) consist of the group sent both a pen and £5 (n = 203), and the group sent £5 only (n = 206). These are compared with the potential participants who were not sent £5 (n = 409), consisting of the group who were sent a pen only (n = 206), and the group who were sent neither a pen nor £5 (n = 203).

**Table 1.** Number of participants randomised to each group.

	Pen	No pen	Total
**£5**	203	206	409
**No £5**	206	203	409
**Total**	409	409	818

**Figure 2.**
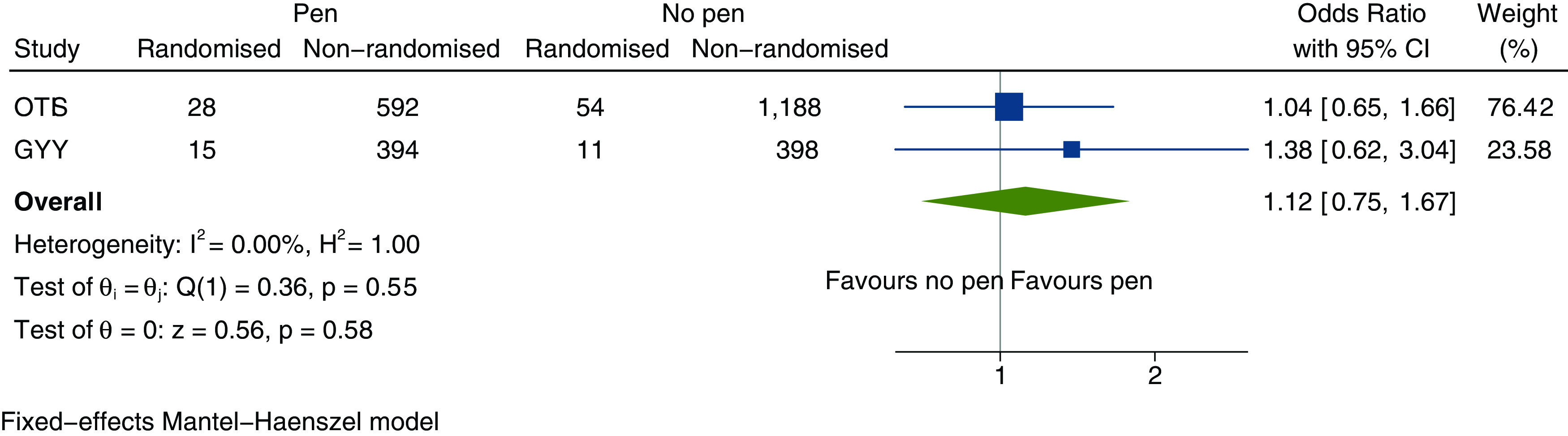
Meta-analysis of inclusion of a pen in postal recruitment packs on randomisation into host trial.

26 (3.2%) SWAT participants were randomised into the host trial ( Table 2). There was no evidence that randomisation rates were improved by including a pen (pen: 15/409, 3.7%; no pen: 11/409, 2.7%; OR 1.38, 95% CI 0.63–3.04, p = 0.43) or £5 (£5: 14/409, 3.4%; no £5: 12/409, 2.9%; OR 1.18, 95% CI 0.54–2.57, p = 0.69) in the recruitment packs. The interaction between the interventions was investigated as a secondary analysis and was not found to be statistically significant (interaction coefficient 0.98, 95% CI 0.20–4.79, p = 0.98). In a meta-analysis with the OTIS SWAT, the pooled OR associated with receipt of a pen was 1.12 (95% CI 0.75–1.67, p = 0.58) ( Figure 2).

**Table 2.** SWAT results.

	Pen (n = 409)	No pen (n = 409)	£5 (n = 409)	No £5 (n = 409)	Interaction coefficient (95% CI), p-value ^c^
**Returned screening form, n (%)**	66 (16.1)	61 (14.9)	77 (18.8)	50 (12.2)	1.66 (0.76–3.60), 0.20
Adjusted odds ratio ^a^ (95% CI), p-value	1.10 (0.75–1.61), 0.61		1.67 (1.13–2.45), 0.01		
**Time to return (days)** ^**b**^ **, median (IQR)**	11 (9–14)	11 (7–18)	11 (10–18)	8.5 (7–14)	1.56 (0.76–3.19), 0.22
Adjusted hazards ratio ^a^ (95% CI), p-value	1.09 (0.77–1.55), 0.61		1.56 (1.09–2.22), 0.02		
**Randomised, n (%)**	15 (3.7)	11 (2.7)	14 (3.4)	12 (2.9)	0.98 (0.20–4.79), 0.98
Adjusted odds ratio ^a^ (95% CI), p-value	1.38 (0.63–3.04), 0.43		1.18 (0.54–2.57), 0.69		

^a^ All comparisons are between the intervention compared with its respective control; treatment effect estimates >1 represent a favourable outcome for the relevant intervention.

^b^ Median and inter-quartile range (IQR) calculated for returned forms only.

^c^ Interaction between the two interventions.

There was no evidence that including a pen increased the likelihood of returning a screening form (pen: 66/409, 16.1%; no pen: 61/409, 14.9%; OR 1.10; 95% CI 0.75–1.61, p = 0.61), but there was strong evidence for including £5 (£5: 77/409, 18.8%; no £5: 50/409, 12.2%; OR 1.67; 95% CI 1.13–2.45, p = 0.01). The interaction between the interventions was investigated as a secondary analysis and was not found to be statistically significant (interaction coefficient 1.66, 95% CI 0.76–3.60, p = 0.20).

**Figure 3.**
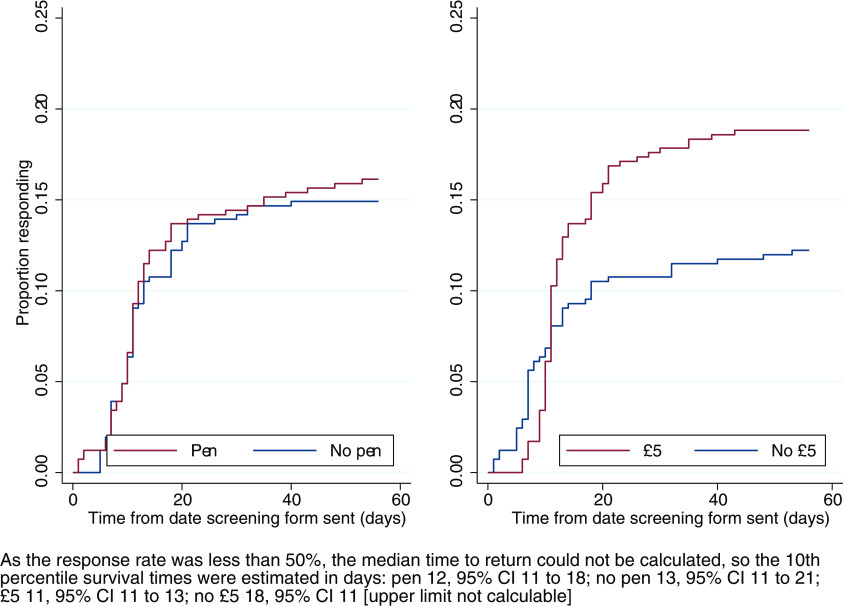
Kaplan–Meier curves for time to return screening form.

There was no evidence of a difference in time to return a screening form associated with inclusion of a pen (HR 1.09; 95% CI 0.77–1.55, p = 0.61), but including £5 decreased the time to return a screening form (HR 1.56; 95% CI 1.09–2.22, p=0.02). See Kaplan–Meier plots ( Figure 3). The Grambsch and Therneau test did not indicate deviation from the proportional hazards assumption. ^3^ The interaction between the interventions was investigated as a secondary analysis and was not found to be statistically significant (interaction coefficient 1.56, 95% CI 0.76–3.19, p = 0.22).

The additional cost of including a pen in the postal mailout was £0.32; the inclusion of £5 additionally cost only the value of the note itself. Given the 1% increase in participants randomised when sent a pen, 100 (1/0.01) potential participants would need to be sent a pen to recruit one additional participant at a cost of £32 (100×£0.32). We would need to send 200 participants £5, at a cost of £1000, to recruit one extra participant.

## Discussion

Participants sent a pen or £5 were marginally more likely to be randomised into the GYY trial than those who did not receive the incentive; however, the differences were not statistically significant. The randomisation rates in the ‘no intervention’ arms (2.7% and 2.9%) were similar to the 3% assumed in the sample size calculation but the observed group differences were smaller than the 4% difference we were powered for; therefore, this SWAT was underpowered to detect the differences observed.

There was little or no evidence that sending a pen increased the likelihood of returning a screening form or decreasing time to return the form.

Although no statistically significant interactions between the pen and £5 were observed, this cannot be ruled out as the sample size of this trial was likely insufficient to be powered to detect an interaction.

A small, monetary incentive was effective at prompting return of the screening form, and of a swifter return, but it did not result in more participants being randomised into the host trial. Some anecdotal evidence from the GYY trial’s process evaluation suggested participants felt it unnecessary to receive £5 with their recruitment pack as they would willingly have joined the trial without this purely to help themselves, others and the research. In addition, this may have caused potential confusion if participants discussed receiving £5 during their yoga sessions as to why some received it and some did not. Such sentiments were not expressed in relation to being sent a pen, potentially suggesting that people view non-monetary incentives differently (more like a gift) than monetary incentives. Since it is relatively costly, we do not recommend this intervention for use to increase recruitment in older adults with multimorbidity.

Pens are cheaper but provided little evidence of benefit. If the observed effect of a 1% difference was true then we would need sufficient SWATs to provide an overall sample size of around 11,000 participants to confirm this. Because the extra cost of recruiting an additional participant is relatively small, more SWATs are required to assess whether this difference is a true effect, since sending pens could be a cost-effective intervention for recruitment.

## Data availability

### Underlying data

OSF: Underlying data for ‘A 2x2 randomised factorial SWAT of the use of a pen and small, financial incentive to improve recruitment rates in a randomised controlled trial of yoga for older adults with multimorbidity’. https://doi.org/10.17605/OSF.IO/2CJZH. ^4^

This project contains the following underlying data:

Data file 1. GYY recruitment SWAT csv data.csv

Data file 2. GYY recruitment SWAT Stata data.dta

Data are available under the terms of the Creative Commons Zero “No rights reserved” data waiver (CC0 1.0 Public domain dedication).

### Reporting guidelines

OSF: CONSORT checklist for ‘A 2x2 randomised factorial SWAT of the use of a pen and small, financial incentive to improve recruitment rates in a randomised controlled trial of yoga for older adults with multimorbidity’. https://doi.org/10.17605/OSF.IO/EU68F. ^5^

Data are available under the terms of the Creative Commons Zero “No rights reserved” data waiver (CC0 1.0 Public domain dedication).
