# A 2x2 randomised factorial SWAT of the use of a pen and small, financial incentive to improve recruitment in a randomised controlled trial of yoga for older adults with multimorbidity

**DOI:** 10.12688/f1000research.52164.2

**Published:** 2022-03-04

**Authors:** Caroline Fairhurst, Jenny Roche, Laura Bissell, Catherine Hewitt, Jess Hugill-Jones, Jenny Howsam, Camila S Maturana, Belen Corbacho Martin, Shirley-Anne S Paul, Fi Rose, David J Torgerson, Lesley Ward, Laura Wiley, Garry A Tew

**Affiliations:** 1 York Trials Unit, ARRC Building, Department of Health Sciences, University of York, Heslington, York, YO10 5DD, UK; 2 British Wheel of Yoga Qualifications, 25 Jermyn Street, Sleaford, NG34 7RU, UK; 3 Department of Sport, Exercise and Rehabilitation, Northumbria University, Newcastle upon Tyne, NE1 8ST, UK

**Keywords:** study within a trial, pen, financial incentive, recruitment, factorial, randomised controlled trial, older people, multimorbidity

## Abstract

**Background:** Monetary and other incentives may increase recruitment to randomised controlled trials.

**Methods: **2x2 factorial ‘study within a trial’ of including a pen and/or £5 (GBP) in cash with a postal recruitment pack to increase the number of participants randomised into the host trial (‘Gentle Years Yoga’) for older adults with multimorbidity. Secondary outcomes: return, and time to return, of screening form, and the cost per additional participant randomised. Binary data were analysed using logistic regression and time to return using Cox proportional hazards regression.

**Results: **818 potential host trial participants were included. Between those sent a pen (n=409) and not sent a pen (n=409), there was no evidence of a difference in the proportion of participants randomised (15 (3.7%) *versus* 11 (2.7%); OR 1.38, 95% CI 0.63–3.04), in returning a screening form (66 (16.1%) *versus* 61 (14.9%); OR 1.10, 95% CI 0.75–1.61) nor in time to return the screening form (HR 1.09, 95% CI 0.77–1.55). Between those sent £5 (n=409) and not sent £5 (n=409), there was no evidence of increased randomisation (14 (3.4%) *versus* 12 (2.9%); OR 1.18, 95% CI 0.54–2.57), but more screening forms were returned (77 (18.8%) *versus* 50 (12.2%); OR 1.67, 95% CI 1.13–2.45) and there was decreased time to return screening form (HR 1.56, 95% CI 1.09–2.22). No significant interaction between the interventions was observed. The cost per additional participant randomised was £32 and £1000 for the pen and £5, respectively.

**Conclusion: **A small, monetary incentive did not result in more participants being randomised into the host trial but did encourage increased and faster response to the recruitment invitation. Since it is relatively costly, we do not recommend this intervention for use to increase recruitment in this population. Pens were cheaper but did not provide evidence of benefit.

## Introduction

Efficient recruitment to randomised controlled trials (RCTs) is important to achieve the target sample size and statistical power within the planned budget and time frame. Incentives, monetary or otherwise, are sometimes used to increase trial recruitment. ^1^ Financial incentives have been found to increase recruitment by 4% (95% CI -1–8%) in a meta-analysis. ^1^ However, most of the included studies used an incentive of £100, which is larger than publicly funded trials can usually afford. There remains, therefore, uncertainty as to whether financial incentives should be used and, if so, what amount.

Offering a potential participant a gift such as a pen may make them more likely to take up the invitation to enrol in a trial. It is also possible that the convenience of having a pen to hand upon receipt of the invitation may help facilitate a swifter response. However, a previous SWAT conducted by the York Trials Unit evaluated the use of pens as an incentive for recruitment into the OTIS trial of older adults and showed no difference in proportion of participants randomised (pen 4.5%; no pen 4.3%, odds ratio (OR) 1.04, 95% CI 0.65–1.67, p = 0.86), or screened (pen 14.2%, no pen 11.7%, OR 1.25, 95% CI 0.94–1.67, p = 0.12), or in time to return screening form (hazard ratio (HR) 1.23, 95% CI 0.94–1.60, p = 0.13). ^2^ To our knowledge, this is the only previous RCT to evaluate pens to increase trial recruitment, so more evidence was needed. We conducted a methodological ‘study within a trial’ (SWAT) to evaluate the effects of including a small, unconditional financial incentive and/or a pen in the postal recruitment pack on the proportion of participants randomised into the host trial. We hypothesised that receipt of a pen or financial incentive would improve the response to the trial invite, encourage a faster response and ultimately result in more participants being randomised into the host trial. We expected an additive effect of receiving both incentives, and that they would not interact.

## Methods

### Design

This 2x2 randomised factorial SWAT was embedded in the Gentle Years Yoga (GYY) trial, which is a multi-centre RCT of the clinical and cost-effectiveness of a yoga programme for older adults with multimorbidity conducted in England (recruitment complete and trial in follow-up at the time of writing; ISRCTN13567538, registered 18/03/2019 https://www.isrctn.com/ISRCTN13567538). The SWAT was registered with the Northern Ireland Network for Trials Methodology Research SWAT repository on 01/04/2018 ( SWAT94; https://www.qub.ac.uk/sites/TheNorthernIrelandNetworkforTrialsMethodologyResearch/SWATSWARInformation/Repositories/SWATStore/). The GYY trial, and its embedded sub-studies, received approval from the North East–York Research Ethics Committee on 24/04/2019 (19/NE/0072), and the Health Research Authority.

### Participants

In order to identify potential participants for the GYY host trial, we asked General Practitioners (GPs) to screen their practice lists for patients who may be eligible and to mail them a recruitment pack. The first 850 patients (see Sample size and randomisation section) mailed a recruitment pack, as identified by four participating GP practices, were included in this SWAT. The standard GYY recruitment pack contained an invitation letter, participant information sheet (PIS), consent and contact details form, screening form, and prepaid envelopes to return documentation to the York Trials Unit, University of York. A random sample of packs additionally included a financial incentive and/or a pen as part of this SWAT. For these packs, the PIS included the following text:

*Please find enclosed a complimentary £5 AND/OR pen given as a thank you for considering taking part. If you choose not to take part you can still keep this.*

The packs were sent out in August 2019.

Potential participants for the host trial were not informed in advance that they were to be sent a recruitment pack for the GYY trial. They were hence also not informed in advance about the SWAT being embedded in the host trial, i.e. that they may receive a pen or some money in the recruitment pack, and that the incentive they received (if allocated to receive a pack containing one) had been chosen through a process of randomisation. (However, as explained above, the PIS, when received, did reference that the pack included £5 and/or a pen as a thank you for considering taking part in GYY.) This means that specific consent for the SWAT was not obtained; this was approved by the Research Ethics Committee as it was considered low risk. As described above, written informed consent for the GYY main trial *was* obtained from all participants who took part.

### Interventions

*Financial incentive*

A £5 (GBP cash) note was enclosed within the recruitment pack for the host trial. The amount of £5 was chosen as we wanted to include a note, rather than coins, as they are lighter (and so did not add any additional postage costs). £5 is the smallest denomination of GBP note; this was deemed sufficient as a thank you to participants for considering taking part in GYY, and including anything larger, e.g. £10, was less likely to be cost-effective.

*Pen*

**Figure 1.**
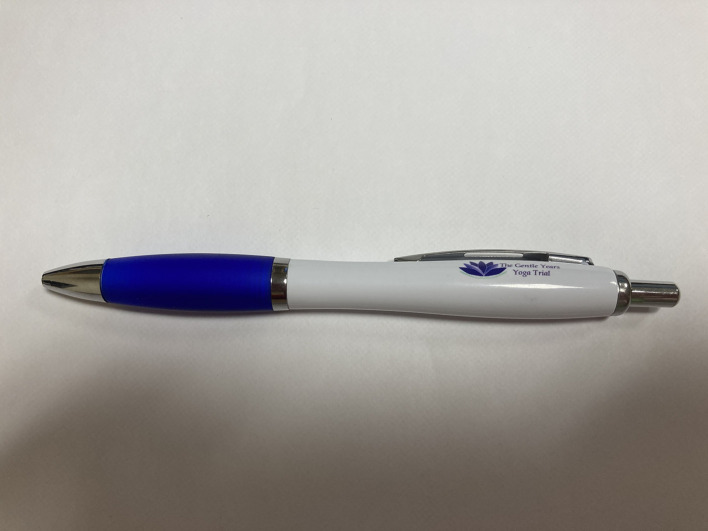
GYY SWAT Pen.

A retractable ballpoint, black ink pen, branded with the GYY trial logo, was enclosed within the recruitment pack for the host trial ( Figure 1).

*Financial incentive + pen*

Both a pen and the cash note were enclosed within the recruitment pack for the host trial.

*Control*

The host trial recruitment pack was sent without the inclusion of a pen or cash.

### Outcome measures

The primary outcome was the proportion of participants randomised into the GYY host trial. Secondary outcomes were return, and time to return, of a screening form to the York Trials Unit, and the cost per additional participant recruited. This was calculated by working out the additional cost of each incentive (i.e. what enclosing a pen or the £5 in cash cost in addition to mailing the standard recruitment pack), and multiplying this by the ‘number needed to treat’ (NNT). The formula for NNT is 1/the absolute risk reduction (ARR). For example, in this case, if the proportion of participants randomised among those who received a pen is X%, and the proportion randomised among those who did not receive a pen is Y%, then the ARR is (X-Y)*100, and the number of people we would need to send a pen to achieve one extra randomised participant is 1/(X-Y)*100.

### Sample size and randomisation

Due to financial restrictions, we could afford to involve a sample of 850 recruitment packs in this SWAT. This would give 80% power (two-sided α=0.05) to detect a difference in the proportion of participants randomised of 4% (from 3% to 7%) for either of the interventions, relative to not receiving that intervention.

Block randomisation of size 4 was used to allocate recruitment packs 1:1:1:1 to: no pen or £5; £5 only; pen only; or pen and £5. A trial statistician, not involved in the production of recruitment packs or recruitment of participants, generated the sequence using Stata v15 (RRID: SCR_012763). Stata is a proprietary software but an open-access alternative in which the sequence could have been generated is Microsoft Excel (RRID: SCR_016137).

### Blinding

Neither the participants nor statisticians analysing the data were blinded to allocation.

### Statistical analysis

The primary comparisons in this trial are the main effects of being sent a pen, and of being sent £5. Returning a screening form and being randomised into the GYY trial were both analysed using multivariable logistic regression, including the two interventions (pen and £5). Time to return the screening form (in days from the date the recruitment pack was sent out to the date it was returned) was analysed using Cox proportional hazards regression. Screening forms that were not returned were censored at eight weeks after they were sent out. These analyses provide an estimate of the average effect of each intervention, assuming there is no interaction between them. In secondary analyses, the interaction between the two interventions was tested by extending the original models to include the interaction term. Analyses were conducted in Stata v16 (RRID: SCR_012763). An open-access alternative that can perform an equivalent function to Stata for analysis is R, a free software environment for statistical computing and graphics (RRID: SCR_001905).

## Results

**Figure 2.**
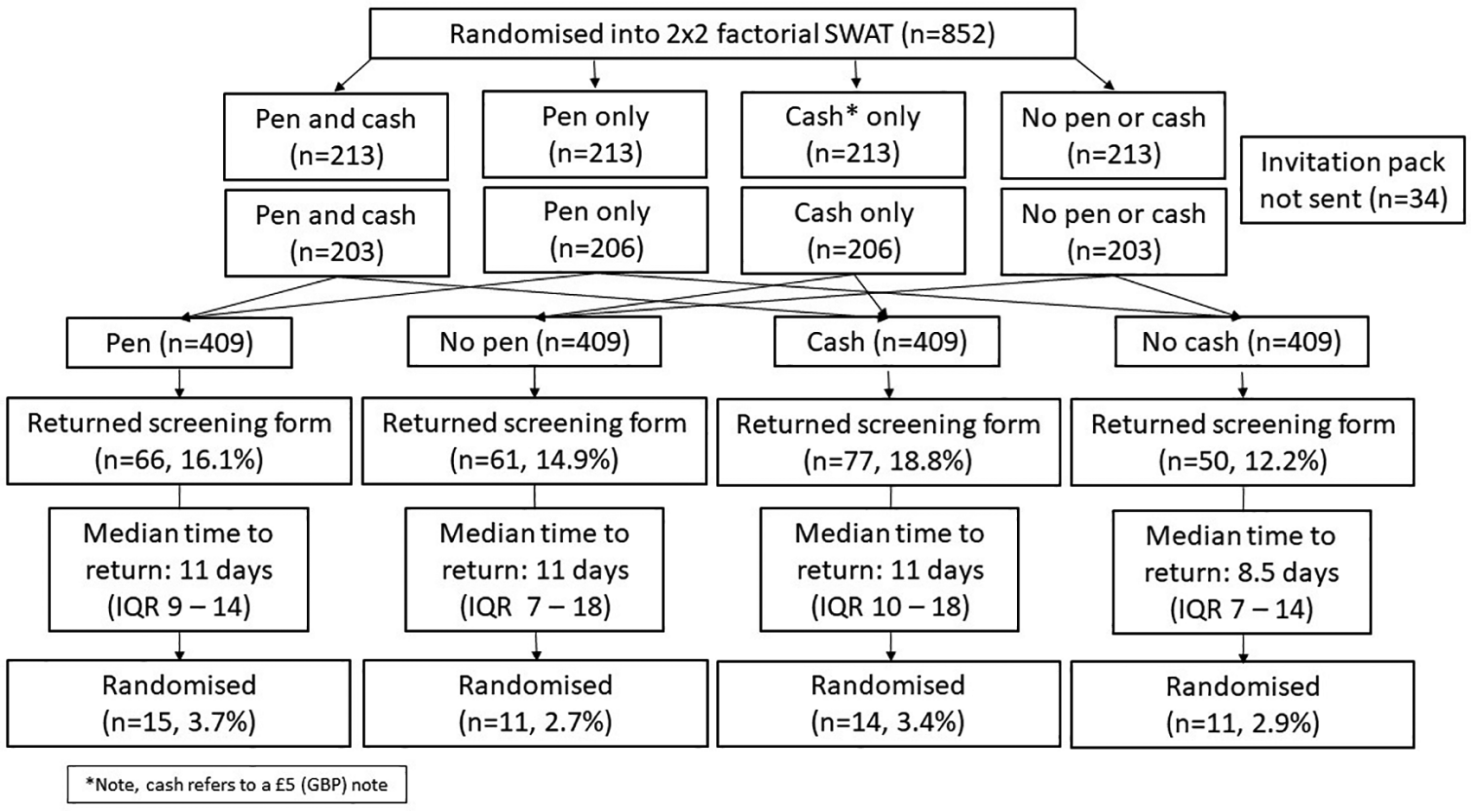
Participant flow diagram.

In total, 852 allocations were generated but, due to one of the participating GP practices having a shorter mailing list than anticipated, only 818 (96.0%) were used ( Table 1; Figure 2).

**Table 1.** Number of participants randomised to each group.

	Pen	No pen	Total
**Cash**	203	206	409
**No cash**	206	203	409
**Total**	409	409	818

Cash refers to a £5 GBP note.

Twenty-six (3.2%) SWAT participants were randomised into the host trial ( Table 2). There was no evidence that the proportion of participants randomised was increased by including a pen (pen: 15/409, 3.7%; no pen: 11/409, 2.7%; OR 1.38, 95% CI 0.63–3.04, p = 0.43) or £5 (£5: 14/409, 3.4%; no £5: 12/409, 2.9%; OR 1.18, 95% CI 0.54–2.57, p = 0.69) in the recruitment packs. The interaction between the interventions was investigated as a secondary analysis and was not found to be statistically significant (interaction coefficient 0.98, 95% CI 0.20–4.79, p = 0.98).

**Table 2.** SWAT results.

	Pen (n=409)	No pen (n=409)	Cash (n=409)	No cash (n=409)	Interaction coefficient (95% CI), p-value
**Randomised, n (%)**	15 (3.7)	11 (2.7)	14 (3.4)	12 (2.9)	0.98 (0.20–4.79), 0.98
Adjusted odds ratio ^a^ (95% CI), p-value	1.38 (0.63–3.04), 0.43		1.18 (0.54–2.57), 0.69		
**Returned screening form, n (%)**	66 (16.1)	61 (14.9)	77 (18.8)	50 (12.2)	1.66 (0.76–3.60), 0.20
Adjusted odds ratio ^a^ (95% CI), p-value	1.10 (0.75–1.61), 0.61		1.67 (1.13–2.45), 0.01		
**Time to return (days)** ^b^ **, median (IQR)**	11 (9-14)	11 (7-18)	11 (10-18)	8.5 (7-14)	1.56 (0.76–3.19), 0.22
Adjusted hazards ratio ^a^ (95% CI), p-value	1.09 (0.77–1.55), 0.61		1.56 (1.09–2.22), 0.02		

Cash refers to a £5 GBP note.

^a^ All comparisons are between the intervention compared with its respective control; treatment effect estimates >1 represent a favourable outcome for the relevant intervention.

^b^ For returned forms.

There was no evidence that including a pen increased the proportion of participants returning a screening form (pen: 66/409, 16.1%; no pen: 61/409, 14.9%; OR 1.10; 95% CI 0.75–1.61, p = 0.61), but there was strong evidence for including £5 (£5: 77/409, 18.8%; no £5: 50/409, 12.2%; OR 1.67; 95% CI 1.13–2.45, p = 0.01). The interaction between the interventions was investigated as a secondary analysis and was not found to be statistically significant (interaction coefficient 1.66, 95% CI 0.76–3.60, p = 0.20).

**Figure 3.**
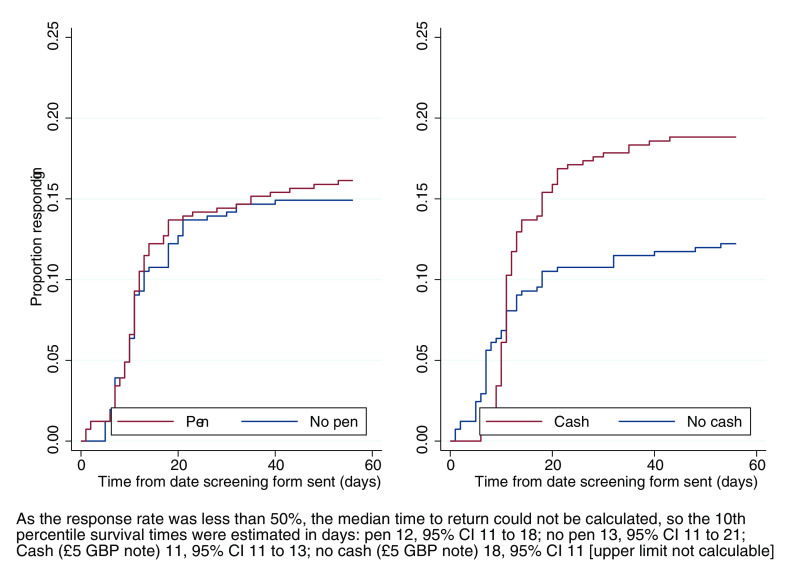
Kaplan–Meier curves for time to return screening form.

There was no evidence of a difference in time to return a screening form associated with inclusion of a pen (HR 1.09; 95% CI 0.77–1.55, p = 0.61), but including £5 decreased the time to return a screening form (HR 1.56; 95% CI 1.09–2.22, p=0.02). See Kaplan–Meier plots ( Figure 3). The Grambsch and Therneau test did not indicate deviation from the proportional hazards assumption. ^3^ The interaction between the interventions was investigated as a secondary analysis and was not found to be statistically significant (interaction coefficient 1.56, 95% CI 0.76–3.19, p = 0.22).

The additional cost of including a pen in the postal mailout was £0.32; the inclusion of £5 additionally cost only the value of the note itself. Given the 1% increase in participants randomised when sent a pen, 100 (1/0.01) pens would need to be sent to recruit one additional participant at a cost of £32 (100×£0.32). Given the 0.5% increase in participants randomised when sent £5, we would need to send 200 participants £5, at a cost of £1000, to recruit one extra participant ((1/0.005)*5).

## Discussion

There was no evidence of a difference in the proportion of participants randomised into the host trial between those sent a pen or £5 and those who did not receive the respective incentive. The proportions of participants randomised in the ‘no intervention’ arms (2.7% and 2.9%) were similar to the 3% assumed in the sample size calculation but the observed group differences were smaller than the 4% difference we were powered for; therefore, this SWAT was underpowered to detect the differences observed.

There was little or no evidence that sending a pen increased the proportion of participants returning a screening form or decreased time to return the form.

**Figure 4.**
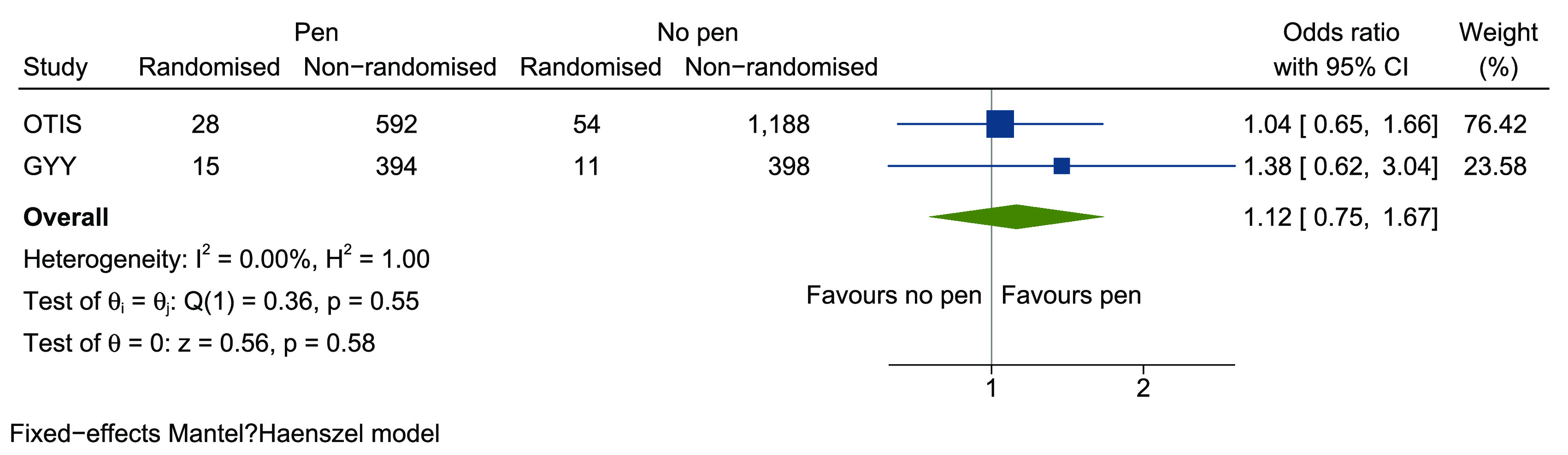
Meta-analysis of inclusion of a pen in postal recruitment packs on randomisation into host trial.

A small, monetary incentive did not result in more participants being randomised into the host trial but was effective at prompting return of the screening form, and of a swifter return. Since sending a financial incentive (even a small one) is, by its very nature, relatively costly, we do not recommend this intervention for use to increase recruitment in older adults with multimorbidity. The pen was cheaper but provided little evidence of benefit. If the observed effect of a 1% difference was true then we would need sufficient SWATs to provide an overall sample size of around 11,000 participants to confirm this. In a meta-analysis with the OTIS SWAT, the pooled OR associated with receipt of a pen was 1.12 (95% CI 0.75–1.67, p = 0.58) ( Figure 4). Because the extra cost of recruiting an additional participant is relatively small, more SWATs are required to assess whether this difference is a true effect, since sending pens could be a cost-effective intervention for recruitment.

Although no statistically significant interactions between the pen and £5 were observed, as was expected, this cannot be ruled out as the sample size of this trial was likely insufficient to be powered to detect an interaction.

For both the pen and cash, the same wording on the participant information sheet was used, which stated that the pen and/or £5 were complimentary and sent as a thank you for people considering taking part in the host trial. Some anecdotal evidence from the GYY trial’s process evaluation suggested that participants felt it unnecessary to receive £5 with their recruitment pack as they would willingly have joined the trial without this purely to help themselves, others and the research. In addition, this may have caused potential confusion if participants discussed receiving £5 during their yoga sessions as to why some received it and some did not. Such sentiments were not expressed in relation to being sent a pen, potentially suggesting that people view non-monetary incentives differently (more like a gift) than monetary incentives.

We were unable to obtain views on receiving the incentives from people sent a recruitment pack but whom either did not return it, were ineligible or declined participation in the GYY host trial. Future SWATs may want to consider ways to obtain qualitative accounts from such participants, as this would add further useful context in which to interpret the findings.

In conclusion, we did not find evidence that the inclusion of a pen and/or £5 was particularly effective or represented good value for money for improving recruitment into a trial of Gentle Years Yoga for older adults with multimorbidity.

## Data availability

### Underlying data

OSF: Underlying data for ‘A 2x2 randomised factorial SWAT of the use of a pen and small, financial incentive to improve recruitment rates in a randomised controlled trial of yoga for older adults with multimorbidity’. https://doi.org/10.17605/OSF.IO/2CJZH. ^4^

This project contains the following underlying data:

Data file 1. GYY recruitment SWAT csv data.csv

Data file 2. GYY recruitment SWAT Stata data.dta

Data are available under the terms of the Creative Commons Zero “No rights reserved” data waiver (CC0 1.0 Public domain dedication).

### Reporting guidelines

OSF: CONSORT checklist for ‘A 2x2 randomised factorial SWAT of the use of a pen and small, financial incentive to improve recruitment rates in a randomised controlled trial of yoga for older adults with multimorbidity’. https://doi.org/10.17605/OSF.IO/EU68F. ^5^

Data are available under the terms of the Creative Commons Zero “No rights reserved” data waiver (CC0 1.0 Public domain dedication).
