# Supplementary material for: A 2x2 randomised factorial SWAT of the use of a pen and small, financial incentive to improve recruitment in a randomised controlled trial of yoga for older adults with multimorbidity
Source: F1000Res. 2022 Mar 4;10:326. Originally published 2021 Apr 27. [Version 2] doi: 10.12688/f1000research.52164.2 (PMC8994083; doi:10.12688/f1000research.52164.2)
Supplement: Peer review history

# Peer review: Reviewer response for version 2

**DOI:** 10.5256/f1000research.121842.r126276

**Published:** 2022-04-08

**Authors:** Frances Shiely

**Affiliations:** 1 Trials Research and Methodologies Unit, HRB Clinical Research Facility, Mercy University Hospital, University College Cork, Cork, Ireland

Thank you for the opportunity to review this again. I am happy with the revisions made and you may update the status to approved.

Is the work clearly and accurately presented and does it cite the current literature?

Yes

If applicable, is the statistical analysis and its interpretation appropriate?

Partly

Are all the source data underlying the results available to ensure full reproducibility?

Yes

Is the study design appropriate and is the work technically sound?

Yes

Are the conclusions drawn adequately supported by the results?

Partly

Are sufficient details of methods and analysis provided to allow replication by others?

No

Reviewer Expertise:

Trial Methodology; Epidemiology

I confirm that I have read this submission and believe that I have an appropriate level of expertise to confirm that it is of an acceptable scientific standard.

# Peer review: Reviewer response for version 1

**DOI:** 10.5256/f1000research.55404.r122638

**Published:** 2022-02-24

**Authors:** Frances Shiely

**Affiliations:** 1 Trials Research and Methodologies Unit, HRB Clinical Research Facility, Mercy University Hospital, University College Cork, Cork, Ireland

**Article Summary**

 This was a 2x2 factorial ‘study within a trial’ of including a pen and/or £5 with a postal recruitment pack to improve randomisation rate (primary outcome) into the host Gentle Years Yoga trial in older adults with multimorbidity. The authors conclude that including a small, monetary incentive encouraged an increased and faster response to the recruitment invitation but did not result in more participants being randomised into the host trial.

**Major**

 The major issue is the phrasing of the primary outcome as the rate of randomisation. You didn’t analyse the time to randomisation in your analysis, you analysed the proportion randomised. I believe a better description is the proportion randomised to the host trial or just simply number of participants randomised to the host trial.

 Given my comments, I would remove the term 'rates' from the title also.

 The presentation of secondary outcomes prior to presenting the primary outcome could be described as selective outcome reporting. Please adjust this throughout and present your primary outcome first, including in your tables.

**Minor**

**General Comments**

 Check your tenses throughout. Sometime you say 'was' and sometimes you say 'has'. 'Consist of' should be 'consisted of'. Check the English language also as sometime some sentences are phrased a little unusually or are missing the indefinite article or definite article.

**Abstract**

 '818 potential host trial participants included'. Add 'were included'.

 In your results you say there was no evidence of a difference in the likelihood of being randomised. This is nitpicking a little but I think it is clear to stick to the number or proportion randomised.

 Conclusion of abstract: Again modify your response here and put your primary outcome first.

**Methods Section**

***Design***

 I think you should include information in this section on the consent. I can see it further down. Perhaps reconsider all the smaller headings to bring the information together. This is a very small issue.

***Participants***

 Remove the word 'appeared' from the first sentence as it introduces unnecessary uncertainty around your eligibility criteria.

***Intervention***

 The information you have currently given in the intervention section belongs in the introduction section. In this section, I would expect to see what the intervention was. I would also expect to see what the control group was in this SWAT. I assume it is no incentive, but it should be clearly stated.

***Outcomes***

 Again, consider the primary outcome here. Also, when you state the secondary outcomes, list them all together. The cost per additional participant recruited looks like it was not part of the secondary outcomes as it is in a separate sentence.

**Results**

 Change 'potential participants' to 'participants' as the SWAT is completed. Delete the following text as it is all evident in Table 1 and is actually easier to follow in the table.

 "In these analyses, the potential participants who were sent a pen (n = 409) consist of the group who received a pen and a £5 note (n = 203), and the group who received a pen only (n = 206). These are compared with those who were not sent a pen (n = 409), consisting of the group who were only sent a £5 note (n = 206), and the group who were sent neither a pen nor £5 (n = 203). Similarly, those who were sent £5 (n = 409) consist of the group sent both a pen and £5 (n = 203), and the group sent £5 only (n = 206). These are compared with the potential participants who were not sent £5 (n = 409), consisting of the group who were sent a pen only (n = 206), and the group who were sent neither a pen nor £5 (n = 203)."

**Conclusion**

 Your primary outcome should be reported first.

Is the work clearly and accurately presented and does it cite the current literature?

Yes

If applicable, is the statistical analysis and its interpretation appropriate?

Partly

Are all the source data underlying the results available to ensure full reproducibility?

Yes

Is the study design appropriate and is the work technically sound?

Yes

Are the conclusions drawn adequately supported by the results?

Partly

Are sufficient details of methods and analysis provided to allow replication by others?

No

Reviewer Expertise:

Trial Methodology; Epidemiology

I confirm that I have read this submission and believe that I have an appropriate level of expertise to confirm that it is of an acceptable scientific standard, however I have significant reservations, as outlined above.

# Response

**Published:** 2022-03-01

**Authors:** Caroline Fairhurst

**Affiliations:** York Trials Unit, UK

Author response:

 Thank you for your helpful review. We have amended the text and title to remove ‘rate(s)’ accordingly. Throughout the Abstract, main body of the text, and the tables we have made sure that the primary outcome is presented before the secondary outcomes. The reason we sometimes presented the outcome of 'returned screening form' first was not to be selective around the reporting of outcomes, but rather to reflect the chronological order in which the outcomes occurred (participants first returned their screening forms and, if eligible, were randomised). However, we can see how this might be construed and so have amended the text and tables accordingly so the primary outcome is presented first. We have changed the Abstract according to all your other suggestions too, plus a few others to keep it within the word limit of 300. 

 We agreed that information on consent was better placed in the Participants section, and so moved this detail up from its original place further down the paper. We have moved text from the Intervention section to the Introduction, and have provided a clear description of the four groups of the SWAT in the Intervention section.

 As suggested, we have deleted text in the Results that was also depicted in Table 1. The use of the terminology ‘potential participant’ refers to the fact that the people sent the recruitment pack were only ‘potential’ participants in relation to the host trial, but they were participants in the SWAT, and so we can see the confusion. We have reconsidered the wording throughout.

 In general, the paper has been proof-read and edits made to improve clarity and readability.

 We really appreciate your comments and believe the changes we have made in response to them have really improved the paper. If you have any other suggestions for improvement, we would be happy to receive them.

# Peer review: Reviewer response for version 1

**DOI:** 10.5256/f1000research.55404.r97206

**Published:** 2022-02-11

**Authors:** Stephan Dombrowski

**Affiliations:** 1 University of New Brunswick, Fredericton, NB, Canada

Thank you for the opportunity to review this very interesting 2x2 experimental SWAT study which I enjoyed reading.  The paper is well-written and interesting. Below are some comments, the authors might want to consider for a potential revision of their manuscript.

**Abstract:**

 Minor suggestion is to re-phrase “into the host Gentle Years Yoga trial in older adults with multimorbidity” to “into the host trial Gentle Years Yoga in older adults with multimorbidity”. Separating ‘host’ and ‘trial’ was confusing on the first reading. There are a couple more instances in the paper which might benefit from keeping host trial as one phrase. Maybe ‘in’ should be ‘for’ in this sentence?

 Results first sentence (“818 potential host trial participants included.”) seems ungrammatical.  ‘were included’ perhaps?

 Conclusions: “ *Including a small, monetary incentive encouraged increased and faster response to the recruitment invitation but did not result in more participants being randomised into the host trial. Since it is relatively costly, we do not recommend this intervention for use to increase recruitment in this population*.”. I think this is accurate, but wonder if you want to consider putting the main message first for your primary outcome (numbers randomised), rather than the secondary outcomes of response rate and speed?

 Conclusions: “Pens are cheaper but did not provide evidence of benefit.”. I suppose that depends on the pen that is chosen.  Either phrase in the past (“were cheaper”) or consider omitting. Not sure it adds much to the conclusions.

 Conclusions: the last sentence seems to include very little study-specific information that wouldn’t be true for almost every research study (“Further studies may be required.”) and could be omitted without losing any critical content.

**Introduction:**

 Overall I do like a focused and succinct introduction but wondered whether a little more information was required to contextualise the study some more.  I found a lot of relevant contextual information in the methods section under ‘Interventions’ which I would have expected in the introduction. I wonder if that can be moved up within the manuscript.  Below I suggest additional information for the methods section which outlines the intervention in somewhat more detail.

 Did the authors have any study aims or a priori hypotheses they were testing within the study? If yes then it might be useful to explicitly state these at the end of the introduction.

**Methods**

 Very minor point: Did participants receive multiple ‘prepaid envelopes’ or just one (all intervention material is described in singular except the envelopes)? The reason I am asking is because the intervention was included as part of the whole package.  The more content in the package, and the more complex the response to the invite (e.g. through having to navigate multiple envelopes), the less likely people are to respond and miss the intervention.

 Under intervention, it would be useful to move the current text to the introduction as it provides a rationale for the study, rather than describing the intervention. It would be useful to describe intervention details, including:

- How was the value of 5 pounds decided?
- Was the money provided in cash?
- Why would the pen and the money components interact? Are there any theoretical reason why such an interaction might occur?
- What did patients get told about the money, if anything? This is important as having seemingly randomly a 5er in a research recruitment package might be confusing to people, unless there is some kind of explanation why it was sent. Incentives and rewards typically have an element of contingency attached to them.
- More details on the pen would be useful, as there is a difference between a cheap throw-away pen and a more substantial and durable one. A picture of the pen would be useful. In fact, a picture of the entire online package with all its content would be even better.

Could the authors explain how they calculated the following “The cost per additional participant recruited was calculated for each intervention.”?

 All the Tables and figures have one group designated as pen/no pen, and the other as £5/ No £5. Would it be better to talk about ‘money’ or ‘cash’ for international audiences (i.e. intervention groups = ‘pen/no pen’ and ‘money/ no money), rather than use the label which designates the exact value in British currency?

**Results**

 The meta-analysis in the results section is somewhat surprising given that it has not been mentioned in the methods section and does not appear to be related to a research question. It might be more useful to compare the results of this study with other similar studies in the discussion section.

**Discussion**

 In line with the conclusions in the abstract, and for consistency of message, I wonder if the authors might want to consider to state no difference in randomisation between the groups, rather than using more ambiguous phrasing of ‘marginally more significant.

 The discussion of the ‘anecdotal evidence’ is interesting, adding qualitative accounts of participants would have added context and additional information useful to interpret the findings. It would have been particularly informative to ask those who returned but declined, as well as those who never returned. Perhaps add a sentence for future SWATs to consider?

 The sentence “Since it is relatively costly, we do not recommend this intervention for use to increase recruitment in older adults with multimorbidity.” could be increased in clarity. The previous paragraph discusses both pen and cash, so it is not clear if the authors mean either or only cash with ‘this intervention’. I assume the money, given the paragraph structure, but a quick read of the paper might lead to some confusion. Replacing ‘this intervention’ with ‘a 5 pounds incentive’ or something might be useful.

 I think that the interpretation of the findings in relation to the incentive depends on how the money was presented in the context of the recruitment package – see methods comments on additional information which would add value to the paper. The conclusion might benefit from taking into consideration that the form of delivery of the incentive could have affected how people saw it, which subsequently impacted their decision making around participation.

 I wonder if a concluding sentence might be beneficial to add to finish the discussion.

Is the work clearly and accurately presented and does it cite the current literature?

Yes

If applicable, is the statistical analysis and its interpretation appropriate?

Yes

Are all the source data underlying the results available to ensure full reproducibility?

Partly

Is the study design appropriate and is the work technically sound?

Yes

Are the conclusions drawn adequately supported by the results?

Yes

Are sufficient details of methods and analysis provided to allow replication by others?

Partly

Reviewer Expertise:

Health psychology

I confirm that I have read this submission and believe that I have an appropriate level of expertise to confirm that it is of an acceptable scientific standard.

# Response

**Published:** 2022-03-01

**Authors:** Caroline Fairhurst

**Affiliations:** York Trials Unit, UK

Author response:

 Thank you for your thorough and constructive review. We have changed the Abstract according to all your suggestions, plus a few others to keep it within the word limit of 300. We have moved text from the Intervention section to the Introduction, where we agree it is better placed, and have added in our hypotheses to the end of the Introduction. In relation to the content of the recruitment packs, all potential participants of the host trial, GYY, received the same standard recruitment pack in the post. This included an invitation letter, participant information sheet (PIS), consent and contact details form, screening form and two prepaid envelopes. The reason that two envelopes were included is that, if the person wished to take part in the trial, we asked them to return their consent form and contact details in one envelope, and their completed screening form in the other (all documents were linked by a unique, prepopulated trial ID number). This is so that, should the post be intercepted, an individual’s contact details are separated from the confidential information they provide in the screening form. In the SWAT, for those randomised to receive a pen and/or £5, the allocated intervention was enclosed within the standard recruitment pack for the host trial. The only other difference was some wording on the PIS, to include:

*“Please find enclosed a complimentary £5 AND/OR pen given as a thank you for considering taking part. If you choose not to take part you can still keep this.”*

 Therefore, participants in all four groups of the SWAT received the exact same contents in the recruitment pack APART FROM the inclusion of a pen and/or £5 if allocated, and the additional wording in the PIS. 

 In the Intervention section, we have elaborated on the incentives, providing detail about why the value of £5 was chosen and a description of the pen. More detail has also been included on how the 'cost per additional participant recruited' was calculated. We have heeded your suggestion to change £5 to 'cash' in some of the text and tables for international audiences, and have moved the pen meta-analysis to the Discussion.

 We have made changes to the Discussion as per all your suggestions, and added in the following concluding sentence:

*“In conclusion, we did not find evidence that the inclusion of a pen and/or £5 was particularly effective or represented good value for money for improving recruitment into a trial of Gentle Years Yoga for older adults with multimorbidity.”*

 We greatly appreciate your comments and believe the changes we have made in response to them have really improved the paper. If you have any other suggestions for improvement, we would be happy to receive them.
